# Nonlinear relationships between the triglyceride glucose-body mass index and cardiovascular disease in middle-aged and elderly women from NHANES (1999–2018)

**DOI:** 10.1038/s41598-025-95677-5

**Published:** 2025-03-31

**Authors:** Chunxue Li, Qiuxia Lin, Chunli Wan, Lin Li

**Affiliations:** Department of Cardiovascular, The Second Hospital of Jilin University, No.218, Zi Qiang Street, Nanguan Zone, Changchun, 130041 Jilin China

**Keywords:** Cardiovascular diseases (CVD), Women, Triglyceride glucose-body mass index (TyG-BMI), NHNAES, Biomarkers, Cardiology, Medical research, Risk factors

## Abstract

This study aims to investigate the relationship between the triglyceride-glucose body mass index (TyG-BMI) and cardiovascular disease (CVD) among middle-aged and elderly women, using data from the National Health and Nutrition Examination Survey (NHANES) spanning from 1999 to 2018. TyG-BMI was calculated as Ln [fasting triglycerides (mg/dL) × fasting glucose (mg/dL)/2] × BMI. After applying the sampling weights from NHANES, the study sample of 6,343 participants is representative of approximately 59,174,898 American women. We categorized TyG-BMI into quartiles, using Q3 as the reference group. In the crude model, Q4 exhibited a 40% increased odds of CVD (odds ratio [OR] = 1.40, 95% confidence interval [CI] 1.09–1.79, *P* = 0.009). In the fully adjusted model, the increased odds of CVD for Q4 relative to Q3 was 39% (OR = 1.39, 95% CI 1.06–1.82, *P* = 0.019). Further analysis using restricted cubic splines (RCS) and threshold effect analysis confirmed a nonlinear relationship between them. Below a TyG-BMI threshold of 260, there was no significant association with CVD odds. However, above 260, each 10-unit increase in TyG-BMI was associated with a 2.4% increase in CVD odds in this demographic. Sensitivity analyses confirmed the stability of the results.

**Supplementary Information:**

The online version contains supplementary material available at 10.1038/s41598-025-95677-5.

## Introduction

Although the incidence of cardiovascular disease (CVD) in women is comparable to that of men approximately ten years younger, this disparity narrows with advancing age. The incidence and mortality rates associated with CVD in women significantly increase around the time of menopause compared to their reproductive years^1,2^. CVD are the leading cause of mortality in women, with 8.9 million female deaths attributed to CVD globally in 2019^3,4^.

Insulin resistance (IR), characterized by reduced sensitivity to insulin, serves as a pathogenic determinant and a predictive biomarker for CVD across diverse populations, including those with diabetes mellitus^5^. The euglycemic insulin clamp and intravenous glucose tolerance test are recognized as the gold standards for assessing insulin resistance (IR). However, their use in routine clinical settings is restricted due to their complexity and cost, despite being employed in academic research. The Homeostasis Model Assessment of Insulin Resistance (HOMA-IR) index stands as a widely recognized tool for evaluating β-cell function and IR, nonetheless, its applicability is limited in patients receiving insulin treatment and in those with non-functional β-cells^6^. As early as 2011, Vasques et al. highlighted that the TyG index has been evaluated as an alternative approach for estimating IR, they noted that the TyG index correlates with markers of adiposity, metabolism, and atherosclerosis associated with IR, and shows a moderate level of concordance with the hyperglycemic clamp technique^7^. Subsequently, TyG have been shown to replace HOMA-IR in IR diagnosis by many studies^8,9^In recent years, a substantial body of research has confirmed the association between the TyG index and the risk of CVD as well as the risk of cardiovascular mortality^10–13^. The triglyceride glucose index (TyG) and triglyceride glucose-body mass index (TyG-BMI) have emerged as a direct and potent alternative indicator for the early detection of IR used to determine CVD risk^5,14,15^. Numerous studies have demonstrated a reliable association between TyG-BMI and conditions such as non-alcoholic fatty liver disease, metabolic syndrome, diabetes, and ischemic stroke^16–19^. A study utilizing the data from National Health and Nutrition Examination Survey (NHANES) has concluded that the TyG-BMI index, serving as a marker for IR, exhibits a significantly positive correlation with the elevated prevalence of CVD^20^. A recent investigation from China has uncovered a significant linear correlation between the cumulative average TyG-BMI index and the incidence of CVD within the middle-aged and elderly demographic (P for overall trend = 0.038, P for nonlinearity = 0.436)^21^. However, there is no consensus on whether gender influences the relationship between TyG-BMI and CVD^20–23^. The relationship between TyG-BMI and CVD in women, especially middle-aged and elderly women, is unclear, and it is meaningful to further explore the relationship.

Therefore, we utilized data from the NHANES (1999–2018) to explore the association between TyG-BMI and CVD in middle-aged and elderly American women.

## Materials and methods

### Data source and study population

NHANES orchestrated by the National Center for Health Statistics (NCHS), serves as a pivotal and nationally representative initiative in the United States. Its purpose is to provide a detailed assessment of the health and nutritional status among the civilian, non-institutionalized demographic. Characterized by a multistage, stratified sampling design that incorporates probability selection, NHANES ensures a comprehensive and diverse participant representation. It has been conducted continuously since 1999, with data collected and released in two-year cycles. Researchers globally may access NHANES data through the survey’s official portal^24^. Furthermore, the need for additional approval from institutional review boards is obviated when conducting secondary analyses^25^. In our study, we strictly followed the guidelines provided by the Strengthening the Reporting of Observational Studies in Epidemiology (STROBE) initiative, which are detailed on their official website^26^.

**Fig. 1:**
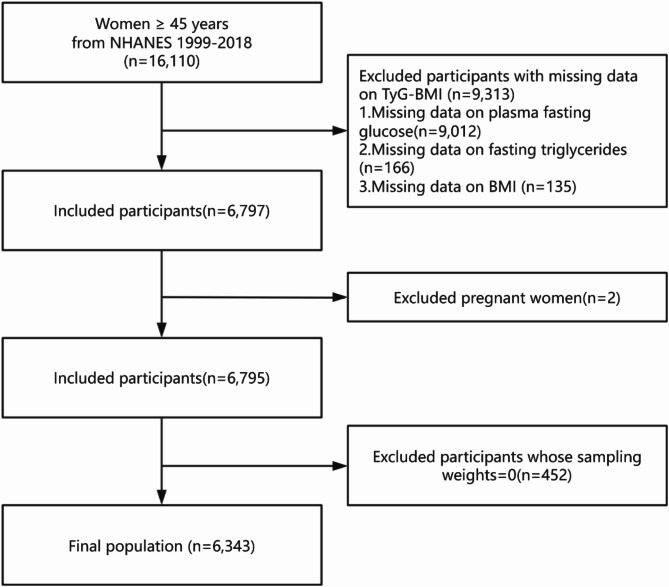
Flowchart depicting the participants’ selection.

In this cross-sectional analysis, we enrolled 6343 women aged 45 years and above from the NHANES between 1999 and 2018. Our selection criteria excluded individuals with incomplete fasting glucose, fasting triglyceride, and BMI records, as well as those who were pregnant or had a sampling weight of zero. The final study population is depicted in Fig. 1.

### Definition of TyG-BMI

The TyG-BMI index was computed utilizing the subsequent formulas: (1) TyG = Ln [fasting triglycerides (mg/dL) × fasting glucose (mg/dL)/2]; (2) BMI = body mass (kg)/height^2^ (m^2^); TyG-BMI = TyG × BMI^20^. Participants were subsequently categorized into four quartile groups (Q1, Q2, Q3, and Q4) based on their TyG-BMI index values.

### Definition of CVD

The identification of CVD was based on self-reported diagnoses provided by participants during personal interviews, utilizing a standardized medical inquiry. Respondents were queried, “Has a doctor or other health expert ever informed you that you have congestive heart failure/coronary heart disease/angina pectoris/myocardial infarction/stroke?” Affirmative responses to any of these queries were indicative of CVD status^10^.

### Clinical data and laboratory tests

Based on existing literature and clinical judgment, we also collected other variables, including age, race/ethnicity, marital status, education level, family income, smoking status, alcohol use, physical activity, diabetes, hypertension, high cholesterol, low density lipoprotein-cholesterol (LDL-C), high density lipoprotein-cholesterol (HDL-C), cancer or malignancy, postmenopausal, number of pregnancies, Metabolic syndrome and HbA1c. Age, LDL-C, HDL-C, HbA1c were recorded as continuous variables. Race/ethnicity was categorized into five distinct groups: Mexican, non-Hispanic black, non-Hispanic white, other Hispanic, and others. Marital status was classified as either being married or cohabiting, and living singly. Education was stratified into three tiers based on years of schooling: less than high school (< 9 years), high school or equivalent (9–12 years), and above high school > 12 years). Family income was gauged against the poverty income ratio (PIR), with designations of low income (PIR ≤ 1.3), medium income (1.3 < PIR ≤ 3.5), and high income (PIR > 3.5). Smoking status were categorized into never smokers, former smokers, and current smokers. Alcohol use was self-reported and divided into five levels: never, former, mild, moderate, and heavy drinkers^27^. We assessed physical activity (PA) levels among participants by determining the total metabolic equivalent of task (MET) minutes per week, which was derived from self-reported data on PA intensity, duration, and frequency over a 1-week period^28^.Diabetes was ascertained by physician diagnosis reported by participants, utilization of antidiabetic medications or insulin, or fasting glucose levels of 126 mg/dL or higher, oral glucose tolerance test results above 200 mg/dL, or glycohemoglobin levels above 6.5%. Hypertension was identified through self-reported physician diagnoses, antihypertensive medication use, or blood pressure measurements exceeding 140 mmHg systolic or 90 mmHg diastolic. High cholesterol was defined by participant reports of high cholesterol, use of lipid-lowering medications, or total cholesterol levels of 240 mg/dL or greater. Cancer or malignancy was based on self-reported diagnoses provided by participants during personal interviews. Individuals who had not experienced menstruation in the past 12 months due to menopause were classified as postmenopausal. The number of pregnancies was ascertained through self-report and categorized into four groups: 0, 1–5, 6–10, and ≥ 10. Metabolic syndrome was diagnosed according to the harmonized criteria^29^. We conducted a screening of covariates (Supplementary Table 1) and ultimately identified age, family income, smoking status, alcohol use, physical activity, diabetes, hypertension, high cholesterol, LDL-C, HDL-C, and HbA1c as the variables for adjustment.

In the execution of our statistical analyses, we accounted for the intricate, multi-stage stratified probability survey design characteristic of NHANES by incorporating sample weights, clustering, and stratification effects into our calculations. This approach ensures that our findings are representative and accurately reflect the complexities of the survey’s sampling framework. We specifically selected the fasting subsample MEC weight as sample weight, and all analyses in our study took these weights into consideration.

### Statistical analysis

Categorical variables were expressed as counts (weighted percentages), and normally distributed continuous variables were presented as means with standard errors (SE). Baseline characteristics were summarized according to TyG-BMI quartile groups. To assess differences among groups, we employed one-way analysis of variance for normally distributed data, the Kruskal–Wallis test for skewed distributions, and the chi-square test for categorical variables. To address missing data in covariates, we employed imputation techniques prior to analysis.

Multivariate logistic regression analysis was employed to examine the association between the TyG-BMI index and CVD. Odds ratios (OR) with 95% confidence intervals (CI) were calculated. The TyG-BMI index was entered into the model both as a continuous variable (with ORs calculated for every 10-point increase) and as a categorical variable (stratified into quartiles with Q3 serving as the reference category).

To account for potential confounders and investigate the relationship between TyG-BMI and CVDodds, we conducted analyses using four progressively adjusted models. The initial model provided unadjusted, crude estimates (Model 1). Subsequent models incrementally adjusted for age (Model 2), followed by additional covariates including family income, smoking status, alcohol consumption, and physical activity (Model 3). The final, fully adjusted model (Model 4) included all covariates from Model 3, with further adjustments for diabetes, hypertension, high cholesterol, LDL-C, HDL-C, and HbA1c. Additionally, we employed restricted cubic splines (RCS) to further investigate the potential nonlinear relationship between TyG-BMI and CVD. Then, we used the likelihood ratio test and bootstrap resampling to determine inflection points after fully adjusted model.

We performed the following sensitivity analyses to validate the stability of the results. First, to detect potential interactions, subgroup analyses were performed by age, diabetes, hypertension, and high cholesterol. Second, we excluded participants with missing data for any covariates and subsequently employed multivariable logistic regression analysis to investigate the relationship between TyG-BMI and CVD. Finally, an unweighted logistic regression analysis was conducted on 6795 participants (prior to excluding participants with zero weights) to explore the relationship between the TyG-BMI index and CVD.

Statistical analyses were performed using R 4.2.2 (http://www.R-project.org, The R Foundation) and Free Statistics software version 2.0, and a two-sided P value < 0.05 was considered to indicate statistical significance.

## Results

This study included 6343 middle-aged and elderly women (after weighting, they represent a population of approximately 59,174,898 in the United States) with a mean TyG-BMI of 255.69 (1.42) and a mean age of 60.67 (0.19) years. Within this group, 915 cases (12.26%) of CVD were identified. Table 1 delineates the basic characteristics of participants according to the quartile of TyG-BMI. Individuals with a higher TyG-BMI index tend to exhibit higher fasting glucose, elevated triglycerides, a higher BMI, and a higher incidence of CVD.

**Table 1:** Baseline characteristics of study participants by TyG-BMI quartiles.

Characteristic	TyG-BMI					*P* value
	Total	Q1 (< 211.20 )	Q2 (211.20 ~ 250.74)	Q3 (250.75 ~ 298.71)	Q4 (> 298.71)	
Weighted	59,174,898	17,222,252	14,543,333	13,752,336	13,656,977	
Unweighted	6343	1586	1585	1586	1586	
Fasting glucose (mg/dL)	107.74 (0.53)	96.67 (0.35)	103.69 (0.66)	109.68 (0.91)	124.06 (1.55)	< 0.001
Triglyceride (mg/dL)	131.35 (1.53)	90.21 (1.54)	122.01 (1.95)	150.05 (2.96)	174.35 (4.13)	< 0.001
TyG	8.69 (0.01)	8.27 (0.02)	8.63 (0.02)	8.87 (0.02)	9.09 (0.02)	< 0.001
BMI, (kg/m^2^)	29.30 (0.14)	22.30 (0.07)	26.79 (0.07)	30.96 (0.08)	39.13 (0.19)	< 0.001
TyG-BMI	255.69 (1.42)	184.20 (0.61)	230.61 (0.38)	273.59 (0.45)	354.52 (1.66)	< 0.001
Race/ethnicity						< 0.001
Mexican American	1,019 (5.05%)	125 (2.26%)	267 (5.22%)	304 (6.19%)	323 (7.24%)	
Non-Hispanic black	568 (4.36%)	106 (2.97%)	150 (5.05%)	170 (5.61%)	142 (4.14%)	
Non-Hispanic white	3007 (73.91%)	916 (79.93%)	739 (72.61%)	693 (71.12%)	659 (70.50%)	
Other Hispanic	1236 (10.58%)	205 (5.96%)	296 (10.82%)	335 (12.04%)	400 (14.71%)	
Others	513 (6.09%)	234 (8.88%)	133 (6.30%)	84 (5.05%)	62 (3.41%)	
Age (year) Mean (SE)	60.67 (0.19)	60.11 (0.37)	61.73 (0.30)	61.46 (0.34)	59.44 (0.33)	< 0.001
Marital status						< 0.001
Married or living with a partner	3351 (61.23%)	890 (66.32%)	833 (59.58%)	847 (61.70%)	781 (56.14%)	
Living alone	2933 (38.77%)	677 (33.68%)	736 (40.42%)	729 (38.30%)	791 (43.86%)	
Education level						< 0.001
Less than high school	948 (7.24%)	168 (5.16%)	218 (6.67%)	297 (9.59%)	265 (8.13%)	
High school or equivalent	2429 (36.97%)	524 (29.09%)	628 (39.99%)	615 (39.16%)	662 (41.51%)	
Above high school	2957 (55.78%)	891 (65.76%)	734 (53.35%)	674 (51.25%)	658 (50.37%)	
Family income						< 0.001
Low	1660 (19.05%)	332 (14.99%)	385 (18.63%)	426 (19.29%)	517 (24.46%)	
Medium	2238 (36.25%)	494 (29.18%)	595 (37.96%)	607 (40.51%)	542 (39.11%)	
High	1789 (44.70%)	608 (55.84%)	437 (43.41%)	398 (40.19%)	346 (36.44%)	
Smoking status						0.1
Never	3840 (57.72%)	948 (57.41%)	999 (59.99%)	992 (58.37%)	901 (55.04%)	
Former	1573 (26.79%)	367 (25.04%)	371 (25.63%)	386 (27.57%)	449 (29.44%)	
Current	922 (15.49%)	269 (17.55%)	211 (14.37%)	208 (14.06%)	234 (15.52%)	
Alcohol use						< 0.001
Never	1240 (17.77%)	278 (15.47%)	325 (17.72%)	318 (19.78%)	319 (18.74%)	
Former	1261 (20.68%)	261 (16.29%)	273 (17.54%)	337 (23.42%)	390 (26.90%)	
Mild	1608 (35.69%)	483 (41.11%)	419 (37.46%)	375 (32.12%)	331 (30.47%)	
Moderate	735 (16.93%)	207 (18.96%)	176 (16.13%)	188 (17.26%)	164 (14.90%)	
Heavy	466 (8.93%)	110 (8.17%)	134 (11.15%)	103 (7.42%)	119 (8.99%)	
Physical activity, (MET)	2197.82 (68.03)	2243.83 (125.67)	2293.75 (124.41)	2456.79 (185.85)	1727.84 (117.29)	< 0.001
Diabetes						< 0.001
No	4581 (79.30%)	1400 (92.98%)	1254 (84.59%)	1091 (75.33%)	836 (60.21%)	
Yes	1662 (20.70%)	166 (7.02%)	310 (15.41%)	467 (24.67%)	719 (39.79%)	
Hypertension						< 0.001
No	2430 (45.18%)	833 (61.28%)	656 (47.68%)	539 (38.71%)	402 (28.77%)	
Yes	3902 (54.82%)	749 (38.72%)	929 (52.32%)	1044 (61.29%)	1180 (71.23%)	
High cholesterol						< 0.001
No	2347 (41.50%)	727 (51.37%)	554 (39.88%)	522 (34.93%)	544 (37.47%)	
Yes	3544 (58.50%)	747 (48.63%)	919 (60.12%)	942 (65.07%)	936 (62.53%)	
LDL-C (mg/dL)	121.97 (0.65)	117.23 (1.15)	126.50 (1.17)	124.69 (1.29)	120.38 (1.22)	< 0.001
HDL-C (mg/dL)	60.60 (0.34)	70.54 (0.67)	61.85 (0.54)	56.08 (0.45)	51.28 (0.42)	< 0.001
Cancer or malignancy						0.27
No	5434 (84.19%)	1337 (82.78%)	1353 (83.35%)	1374 (85.57%)	1370 (85.49%)	
Yes	901 (15.81%)	247 (17.22%)	230 (16.65%)	211 (14.43%)	213 (14.51%)	
Postmenopausal						0.004
No	1683 (32.58%)	431 (36.42%)	405 (31.35%)	384 (28.00%)	463 (33.57%)	
Yes	4302 (67.42%)	1067 (63.58%)	1091 (68.65%)	1095 (72.00%)	1049 (66.43%)	
Number of pregnancies						< 0.001
0	456 (8.42%)	144 (10.24%)	114 (7.52%)	91 (7.18%)	107 (8.31%)	
1 ~ 5	4604 (81.08%)	1185 (81.88%)	1156 (82.50%)	1141 (81.30%)	1122 (78.34%)	
6 ~ 10	822 (9.71%)	148 (7.44%)	208 (9.52%)	225 (10.72%)	241 (11.76%)	
> 10	94 (0.80%)	15 (0.45%)	17 (0.46%)	21 (0.79%)	41 (1.59%)	
Metabolic syndrome						< 0.001
No	3185 (54.47)	1474 (93.85)	946 (63.05)	514 (31.82)	251 (18.47)	
Yes	3158 (45.53)	112 (6.15)	639 (36.95)	1072 (68.18)	1335 (81.53)	
HbA1c	5.77 (0.02)	5.45 (0.01)	5.65 (0.03)	5.85 (0.02)	6.23 (0.04)	< 0.001
CVD						< 0.001
No	5428 (87.74%)	1411 (90.45%)	1375 (88.61%)	1366 (87.62%)	1276 (83.52%)	
Yes	915 (12.26%)	175 (9.55%)	210 (11.39%)	220 (12.38%)	310 (16.48%)	

Data are presented as unweighted number (weighted percentage) for categorical variables and mean (SE) for continuous variables.

*TyG-BMI* triglyceride glucose-body mass index, *TyG* triglyceride glucose index, *BMI* body mass index, *SE* Standard error, *MET* metabolic equivalent of task, *LDL-C* low density lipoprotein-cholesterol, *HDL-C* high density lipoprotein-cholesterol, *CVD* cardiovascular disease.

### Association between TyG-BMI and CVD

The multivariate logistic regression analysis (Table 2) disclosed that, in the crude model, there were no statistically significant differences in the odds of CVD within Q1 and Q2 compared to the reference group (Q3), while the Q4 exhibited a 40% increased odds of CVD. This relationship remained largely consistent across different adjustment models. In the fully adjusted model, no statistically significant differences in CVD odds were observed for Q1 and Q2 relative to Q3, whereas the Q4 showed a 39% increase in CVD odds.

**Table 2:** Multivariable logistic regression analysis of TyG-BMI and CVD odds

Variable	Total no.	Event (%)	Model 1		Model 2		Model 3		Model 4	
			OR (95% CI)	P value	OR (95% CI)	P value	OR (95% CI)	P value	OR (95% CI)	P value
TyG-BMI*	6343	915 (12.26)	1.03 (1.02, 1.05)	< 0.001	1.04 (1.03, 1.06)	< 0.001	1.04 (1.02, 1.05)	< 0.001	1.01 (1.00, 1.03)	0.114
TyG-BMI (Quantile)										
< 211.20	1586	175 (9.55)	0.75 (0.55, 1.01)	0.060	0.78 (0.57, 1.05)	0.101	0.84 (0.62, 1.12)	0.235	1.12 (0.81, 1.55)	0.488
211.20 ~ 250.74	1585	210 (11.39)	0.91 (0.71, 1.16)	0.444	0.87 (0.67, 1.14)	0.314	0.90 (0.69, 1.18)	0.434	1.07 (0.81, 1.40)	0.641
250.75 ~ 298.71	1586	220 (12.38)	Reference		Reference		Reference		Reference	
> 298.71	1586	310 (16.48)	1.40 (1.09, 1.79)	0.009	1.7 (1.31, 2.20)	< 0.001	1.61 (1.24, 2.10)	< 0.001	1.39 (1.06, 1.82)	0.019

*For each 10-unit increase in the TyG-BMI.

Model 1 was without covariate adjustment.

Model 2 was adjusted for age.

Model 3 was adjusted for age, family income, smoking status, alcohol use, physical activity.

Model 4 was adjusted for age, family income, smoking status, alcohol use, physical activity, diabetes, hypertension, high cholesterol, LDL-C, HDL-C, and HbA1c.

Data are presented as unweighted number (weighted percentage).

*TyG-BMI* triglyceride glucose-body mass index, *CVD* cardiovascular disease.

### Dose–response curve between TyG-BMI and the odds of CVD

**Fig. 2:**
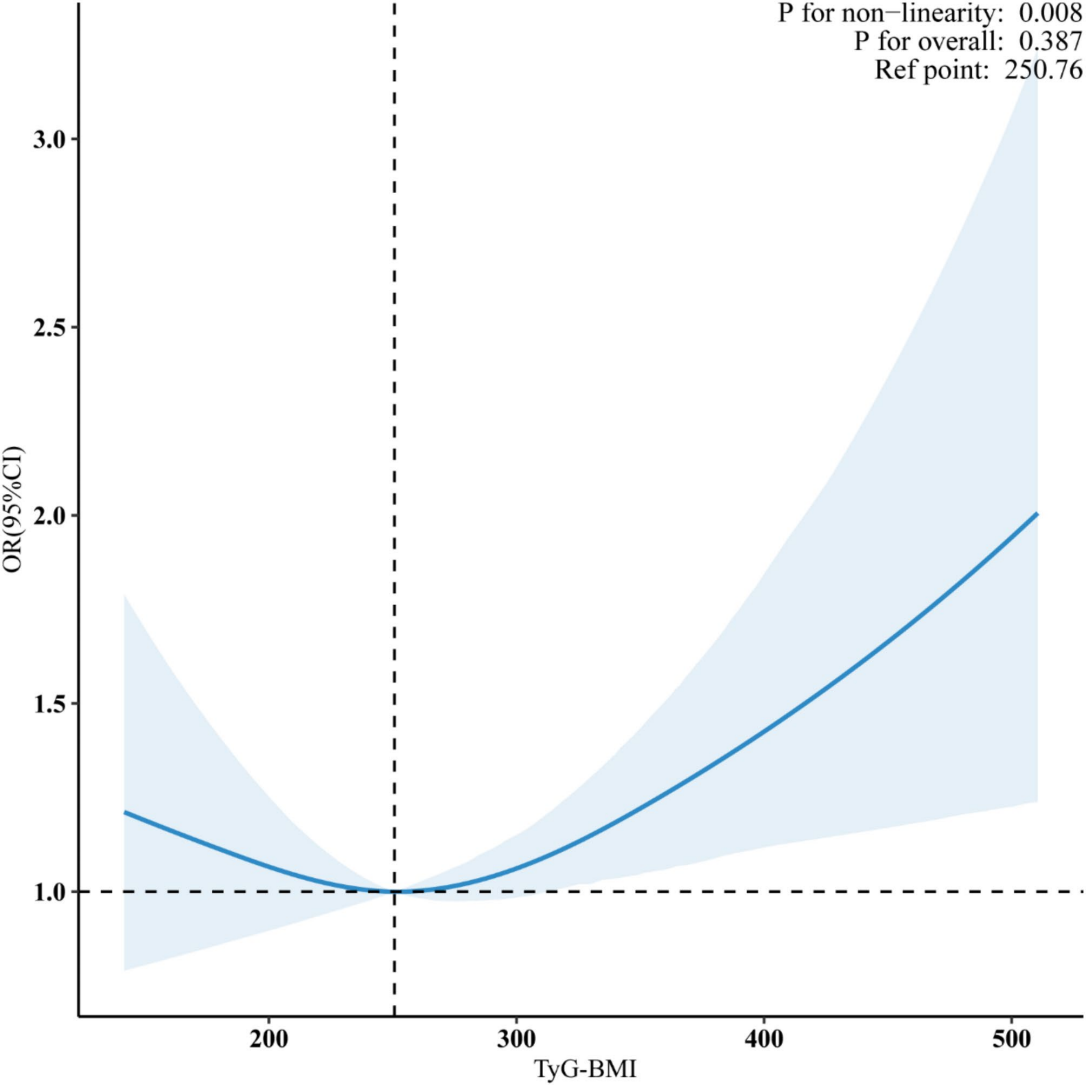
Restricted cubic spline analysis of TyG-BMI and CVD odds.

In this study, RCS were employed to further examine the dose-response relationship between TyG-BMI and the odds of CVD. In the fully adjusted model, a nonlinear relationship emerged between TyG-BMI and the odds of CVD (P for nonlinearity = 0.008) (Fig. 2).Through further threshold effect analysis (Table 3), we identified 260 as the critical threshold in the nonlinear relationship between TyG-BMI and the odds of CVD among middle-aged and elderly women. Below this threshold (TyG-BMI < 260), there was no statistically significant association with the odds of CVD. However, at and above this value (TyG-BMI ≥ 260), an increase in TyG-BMI was correlated with a higher incidence of CVD. For each 10-unit increase in the TyG-BMI, the odds of CVD in this demographic rose by 2.4% (OR = 1.024, 95% CI 1.004, 1.045, *p* = 0.021).

Solid and shaded represent the predicted value and 95% CI. They were adjusted for age, family income, smoking status, alcohol use, physical activity, diabetes, hypertension, high cholesterol, LDL-C, HDL-C, and HbA1c, and 99% of the data is shown.

**Table 3:** Threshold effect analysis of the relationship of TyG-BMI and the odds of CVD.

Threshold of TyG-BMI	OR*	95%CI*	*P* value
< 260	0.985	0.932, 1.040	0.575
≥ 260	1.024	1.004, 1.045	0.021

*For each 10-unit increase in the TyG-BMI.

Adjusted for age, family income, smoking status, alcohol use, physical activity, diabetes, hypertension, high cholesterol, LDL-C, HDL-C, and HbA1c.

### Sensitivity analyses

To ascertain the robustness of our findings, we conducted several sensitivity analyses. First, we performed subgroup analyses stratified by age (< 65 years or ≥ 65 years), diabetes, hypertension, and high cholesterol to identify potential interactions, the P-values for interaction were greater than 0.05 across all groups. These analyses demonstrated that the relationship between TyG-BMI and CVD was stable across various subgroups (Supplementary Fig. 1). Furthermore, after excluding participants with incomplete data for any covariates, the final sample consisted of 2,787 participants. Multivariable logistic regression was used to investigate the association between TyG-BMI and CVD. The results were similar to our initial findings (Supplementary Table 2), and the RCS analysis also showed a similar pattern (Supplementary Fig. 2). Finally, we conducted an unweighted logistic regression analysis on 6,795 participants (prior to excluding participants with zero weights) to explore the relationship between the TyG-BMI index and CVD. The results were consistent (Supplementary Tables 3 and Supplementary Fig. 3). In the sensitivity analysis, to enhance comparability with the primary analysis, we maintained the same grouping values for TyG-BMI that were utilized previously.

## Discussion

Our findings indicate a nonlinear relationship between TyG-BMI and the odds of CVD. Specifically, lower TyG-BMI values were not significantly associated with CVD odds. In contrast, higher TyG-BMI values (> 298.71) were significantly linked to an increased odds of CVD. Further analysis using RCS confirmed that for TyG-BMI > 260, each increment of 10 units in TyG-BMI was associated with a 2.4% increase in the odds of CVD.

The nonlinear relationship between TyG-BMI and CVD has been corroborated in other studies. For example, a study involving participants with prediabetes or diabetes from the NHANES dataset found that individuals with elevated TyG-BMI are at a higher risk of heart failure, with a nonlinear relationship observed (P for non-linearity = 0.014)^30^. Similarly, a prospective cohort study with 355,242 participants from the UK Biobank demonstrated a nonlinear association between elevated TyG-BMI levels and increased risk of sudden cardiac arrest, with higher TyG-BMI indices linked to greater risk and earlier onset, particularly among women^22^. While the RCS trends in these studies align with our findings, neither study provided further analysis of the specific inflection points. In contrast, a study among middle-aged and older Chinese individuals identified a nonlinear relationship between TyG-BMI and stroke risk, with an inflection point at a TyG-BMI value of 174.63^31^. Below this threshold, each 10-unit increase in TyG-BMI was associated with a significant 14.4% increase in stroke risk. Above this inflection point, each 10-unit increase in TyG-BMI corresponded to a smaller 3.8% increase in stroke risk. This contrasts with another study from China, which reported a positive linear association between cumulative average TyG-BMI and CVD incidence (P for overall = 0.038, P for nonlinear = 0.436)^21^. A similar linear association was observed in the Cardiovascular-Kidney-Metabolic CKM syndrome population (P for overall < 0.001, P for nonlinear = 0.355)^23^. Additional studies from NHANES have confirmed that higher TyG-BMI values are significantly associated with an increased prevalence of CVD. Notably, interactions were observed in age subgroups divided at 50 years, with no such association detected in individuals aged 50 years or older (OR = 1.001, 95% CI 1.000-1.002, *P* = 0.069). Similar interactions were observed in the hyperlipidemia subgroup, while results remained stable in the hypertension and diabetes subgroups^20^. Another NHANES study highlighted that the association between TyG-BMI and CVD risk in American adults varies across different glucose metabolic states. Specifically, a U-shaped curve with an inflection point at 270.45 was observed only in individuals with impaired glucose tolerance^32^. In our study, we performed subgroup analyses stratified by age (< 65 years or ≥ 65 years), diabetes, hypertension, and high cholesterol. No significant interactions were detected.

TyG-BMI is not only associated with the risk of CVD, but has also been extensively documented in relation to cardiovascular mortality across numerous studies. However, the protective effect of lower TyG-BMI appears to be absent. For instance, in an NHANES cohort study, a U-shaped relationship was identified between baseline TyG-BMI and CVD mortality among people with diabetes. Specifically, lower TyG-BMI values (< 270.19) were associated with reduced mortality risk (HR 0.64, 95% CI 0.48–0.86), whereas higher TyG-BMI values (> 270.19) were linked to increased risk (HR 1.33, 95% CI 1.06–1.68)^33^. A similar U-shaped curve has been observed in patients with chronic kidney disease^34^. In critically ill patients with atrial fibrillation, lower TyG-BMI levels were significantly associated with increased all-cause mortality at 30, 90, 180, and 365 days^35^. However, the relationship between TyG-BMI and cardiovascular mortality risk in middle-aged and older women remains unclear and warrants further investigation.

CVD exhibits sex differences, with studies indicating that although women have a lower prevalence of CVD compared to men, they are more likely to experience higher mortality rates and poorer prognoses^36^. Additionally, during the menopause transition (MT), the risk of coronary heart disease (CHD) significantly increases. This may be attributed to substantial detrimental changes in various cardiometabolic risk factors during MT, including lipid profiles, vascular health, metabolic syndrome, and visceral adiposity^37^. In our study, we observed that as TyG-BMI increased, several other indicators among middle-aged and older women exhibited significant trends. Specifically, fasting glucose, triglycerides, and BMI—all intrinsic components of the TyG-BMI calculation—increased with higher TyG-BMI quartiles. Concurrently, the prevalence of diabetes, hypertension, and metabolic syndrome also rose with increasing TyG-BMI. HDL-C exhibited a decreasing trend with higher TyG-BMI quartiles, while high cholesterol and LDL-C showed nonlinear patterns. These findings are consistent with those reported by Li et al.^23^ and align with the high-risk profile for CVD incidence. Traditional cardiometabolic risk factors, such as hypertension, diabetes, and dyslipidemia, have long been recognized as significant contributors to CVD. Individuals with metabolic syndrome have a threefold increased risk of coronary heart disease and stroke, with markedly higher cardiovascular mortality^38^. Compared to individuals with a normal BMI, those who are overweight have a significantly higher risk of developing CVD at an earlier age^39^. Education and socioeconomic status also revealed intriguing patterns. The proportion of participants with an education level above high school and those with high income decreased with increasing TyG-BMI, while the proportion of individuals with low income increased. Consistent with previous research findings, individuals with less than a high school education or only a high school diploma have a higher lifetime risk of CVD compared to college graduates^40^. A study utilizing data from NHANES confirmed a positive correlation between the number of pregnancies and an individual’s history of CVD, including coronary heart disease (CHD), congestive heart failure (CHF), angina, heart attack, and stroke^41^. We observed a similar trend, with a higher proportion of women with six or more pregnancies in the higher TyG-BMI groups. Statistically significant differences were observed across quartiles. We also noted interesting trends in other factors. For example, the proportions of the Mexican-American and Other Hispanic groups increased with higher TyG-BMI quartiles, whereas the proportions of non-Hispanic whites and “Others” showed an opposite trend. This warrants further investigation and may be related to lifestyle differences among different ethnic groups.

In our study, we aimed to investigate the association between the TyG-BMI index and CVD odds. To achieve this, we utilized all continuous cycles of NHANES data prior to the COVID-19 pandemic, and adjusting for sampling weights to enhance the representativeness of our study population. We employed multivariate logistic regression analysis, constructing four models with varying covariate adjustments. The robustness of our findings was further assessed through subgroup analyses, as well as sensitivity analyses excluding incomplete participants and non-weighted approaches. Despite these efforts, our study faced several limitations. Firstly, the cross-sectional design of the study did not allow for the establishment of causality. Secondly, although we accounted for numerous potential confounders, the possibility of unmeasured confounders cannot be ruled out. Additionally, the diagnosis of CVD was based on self-report rather than clinical confirmation. Therefore, large-scale prospective cohort studies are necessary to comprehensively evaluate the relationship between TyG-BMI and CVD risk in middle-aged and older women, to elucidate the underlying mechanisms, and to explore potential interventions targeting TyG-BMI as a modifiable risk factor for CVD.

## Conclusions

In summary, this study conclude that there is a nonlinear relationship between TyG-BMI and CVD among middle-aged and elderly women in the United States. When TyG-BMI is ≥ 260, the odds of CVD increases with rising TyG-BMI values. The TyG-BMI index may serve as a cost-effective monitoring indicator and a non-invasive assessment tool in the cardiovascular health management of middle-aged and elderly women.

## Electronic supplementary material

Below is the link to the electronic supplementary material.

Supplementary Material 1

## Data Availability

The National Health and Nutrition Examination Survey (NHANES) data are publicly available at https://www.cdc.gov/nchs/nhanes/.Further inquiries can be directed to the corresponding author.

## Abbreviations

NHANES: National health and nutrition examination survey
CVD: Cardiovascular disease
TyG: Triglyceride glucose
TyG-BMI: Triglyceride glucose-body mass index
BMI: Body mass index
RCS: Restricted cubic spline
SE: Standard error
OR: Odds ratio
CI: Confidence interval
IR: Insulin resistance
LDL-C: Low density lipoprotein-cholesterol
HDL-C: High density lipoprotein-cholesterol
PIR: Poverty income ratio
MET: Metabolic equivalent of task
HOMA-IR: Insulin resistance
MT: Menopause transition
