# Supplementary material for: Nonlinear relationships between the triglyceride glucose-body mass index and cardiovascular disease in middle-aged and elderly women from NHANES (1999–2018)
Source: Sci Rep. 2025 Mar 31;15:10953. doi: 10.1038/s41598-025-95677-5 (PMC11958736; doi:10.1038/s41598-025-95677-5)
Supplement: Supplementary file 1 — Supplementary Material 1 [file 41598_2025_95677_MOESM1_ESM.pdf]

Nonlinear relationships between the triglyceride glucose-body mass index and cardiovascular disease in middle-aged and elderly women from NHANES (1999-2018)

Chunxue Li[a], Qiuxia Lin[a], Chunli Wan[a], Lin Li[a*]

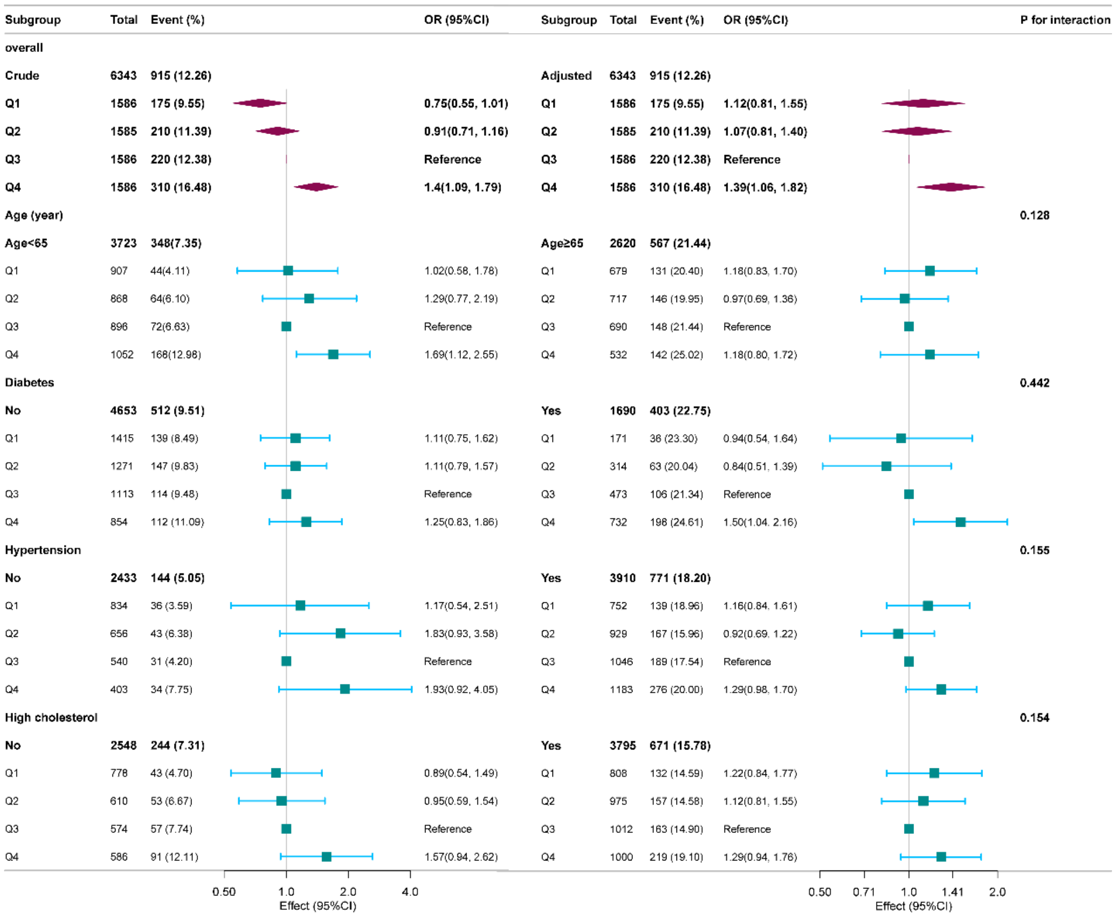

Supplementary Figure 1. Subgroup Analysis for the Association between TyG-BMI and CVD.

For each 10-unit increase in the TyG-BMI

Data are presented as unweighted number (weighted percentage).

They were adjusted for age, family income, smoking status, alcohol use, physical activity, diabetes, hypertension, high cholesterol, LDL-C, HDL-C, and HbA1c.

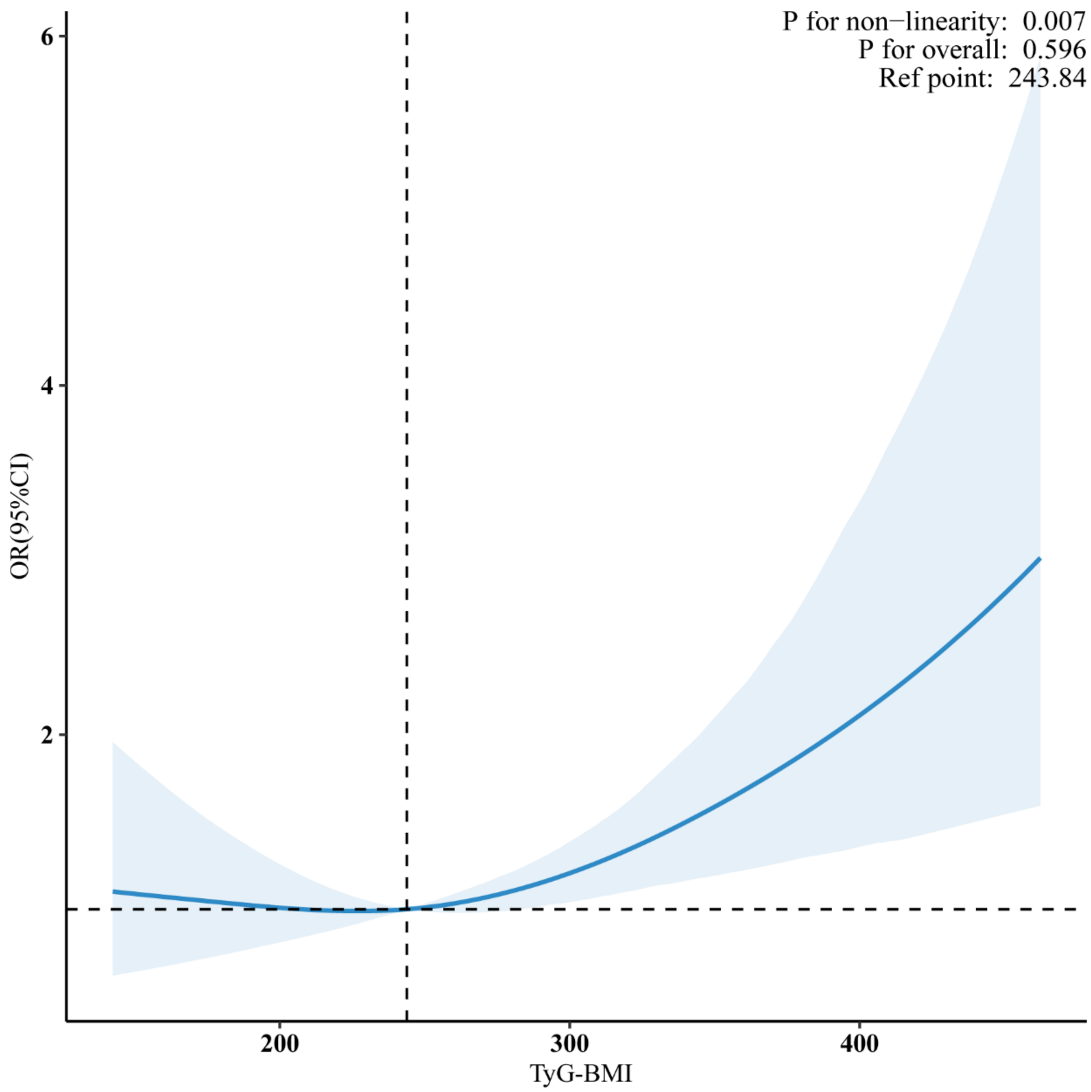

Supplementary Figure 2. Restricted Cubic Spline Analysis of TyG-BMI and CVD Odds (2,787 participants)

Solid and shaded represent the predicted value and 95% CI. They were adjusted for age, family income, smoking status, alcohol use, physical activity, diabetes, hypertension, high cholesterol, LDL-C, HDL-C, and HbA1c, and 99% of the data is shown.

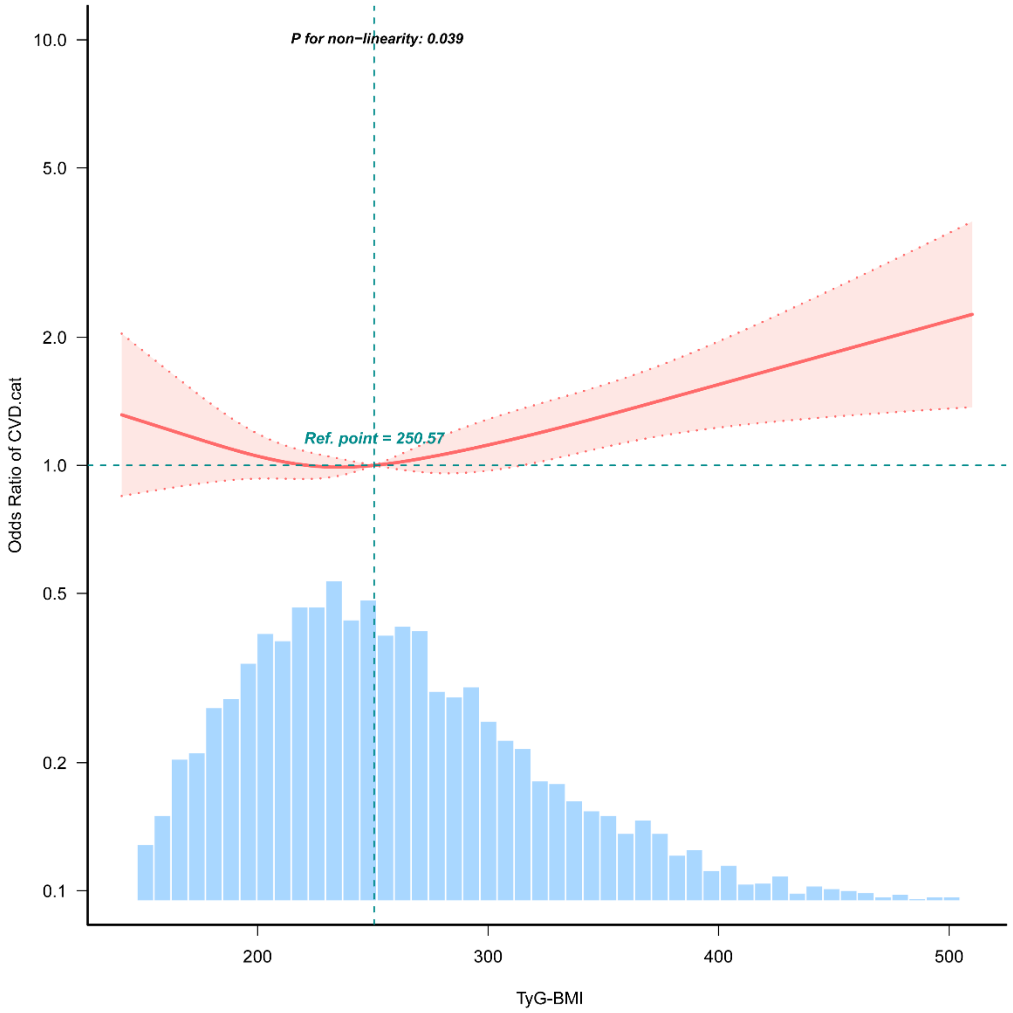

Supplementary Figure 3. Restricted Cubic Spline Analysis of TyG-BMI and CVD Odds (unweighted)

Solid and dashed lines represent the predicted value and 95% CI. They were adjusted for age, family income, smoking status, alcohol use, physical activity, diabetes, hypertension, high cholesterol, LDL-C, HDL-C, and HbA1c, and 99% of the data is show

Supplementary Table 1. Covariate Screening.

Covariate	Coefficient	Change (%)	GVIF	DF	GVIF^(1/(2*Df))	Colinearity	Selection	VIF Selection
Crude	0	Ref.	1.433	1	1.197	0	Ref.	Ref.
Age	0	42.9	1.808	1	1.344	0	Yes	Yes
Race/ethnicity	0	-1.9	1.803	4	1.076	0	No	No
Marital status	0	-3.4	1.175	1	1.084	0	No	No
Education level	0	-5.2	1.652	2	1.134	0	No	No
Family income	0	-16.2	1.477	2	1.102	0	Yes	Yes
Smoking status	0	-1.5	1.534	2	1.113	0	Yes	Yes
Alcohol use	0	-9.6	1.524	4	1.054	0	Yes	Yes
Physical activity	0	25.6	1.108	1	1.053	0	Yes	Yes
Diabetes	0	-52.2	1.29	1	1.136	0	Yes	Yes
Hypertension	0	-38.1	1.117	1	1.057	0	Yes	Yes
High cholesterol	0	-9.2	1.114	1	1.055	0	Yes	Yes
LDL-C	0	-3.1	1.148	1	1.072	0	Yes	Yes
HDL-C	0	-20.9	1.272	1	1.128	0	Yes	Yes
Cancer or malignancy	0	1	1.101	1	1.049	0	No	No
Postmenopausal	0	0.2	1.229	1	1.108	0	No	No
Number of pregnancies.	0	-4.5	1.168	3	1.026	0	No	No
HbA1c	0	-24.4	1.604	1	1.266	0	Yes	Yes

Supplementary Table 2. Multivariable Logistic Regression Analysis of TyG-BMI and CVD Odds (2787 participants)

Variable	Total No.	Event (%)	Model 1		Model 2		Model 3		Model 4	
			OR(95%CI)	P value	OR(95%CI)	P value	OR(95%CI)	P value	OR(95%CI)	P value
TyG-BMI*	2,787	317(9.70)	1.05(1.03, 1.07)	<0.001	1.06(1.04, 1.09)	<0.001	1.06(1.03, 1.08)	<0.001	1.03(1.01, 1.06)	0.018
TyG-BMI(Quantile)										
<211.20	794	68(6.86)	0.79(0.52, 1.22)	0.286	0.82(0.53, 1.27)	0.381	0.89(0.58, 1.37)	0.587	1.18(0.75, 1.85)	0.467
211.20~250.74	717	79(9.82)	1.17(0.78, 1.78)	0.447	1.15(0.74, 1.78)	0.539	1.26(0.79, 1.980	0.327	1.55(1.00, 2.41)	0.051
250.75~298.71	683	66(8.49)	Reference		Reference		Reference		Reference	
>298.71	593	104(15.77)	2.02(1.31, 3.12)	0.002	2.52(1.61, 3.95)	<0.001	2.59(1.64, 4.10)	<0.001	2.27(1.40, 3.69)	0.001

*For each 10-unit increase in the TyG-BMI

Model 1 was without covariate adjustment.

Model 2 was adjusted for age.

Model 3 was adjusted for age, family income, smoking status, alcohol use, physical activity.

Model 4 was adjusted for age, family income, smoking status, alcohol use, physical activity, diabetes, hypertension, high cholesterol, LDL-C, HDL-C, and HbA1c

Data are presented as unweighted number (weighted percentage)

TyG-BMI: triglyceride glucose-body mass index CVD: cardiovascular disease

Supplementary Table 3. Multivariable Logistic Regression Analysis of TyG-BMI and CVD Odds (unweighted)

Variable	Total No.	Event (%)	Model 1		Model 2		Model 3		Model 4	
			OR(95%CI)	P value	OR(95%CI)	P value	OR(95%CI)	P value	OR(95%CI)	P value
TyG-BMI*	6,795	1,027 (15.1)	1.04 (1.03~1.04)	<0.001	1.05 (1.04~1.06)	<0.001	1.06 (1.04~1.08)	<0.001	1.03 (1.01~1.06)	0.002
TyG-BMI(Quantile)										
<211.20	1,704	193 (11.3)	0.76 (0.62~0.93)	0.008	0.73 (0.6~0.9)	0.003	0.81 (0.58~1.14)	0.23	1.21 (0.81~1.8)	0.344
211.20~250.74	1,698	247 (14.5)	1.01 (0.84~1.23)	0.889	0.95 (0.78~1.16)	0.634	1.09 (0.79~1.51)	0.603	1.42 (0.99~2.04)	0.059
250.75~298.71	1,697	244 (14.4)	Reference		Reference		Reference		Reference	
>298.71	1,696	343 (20.2)	1.51 (1.26~1.81)	<0.001	1.82 (1.51~2.2)	<0.001	2.18 (1.59~2.98)	<0.001	2.06 (1.44~2.94)	<0.001

*For each 10-unit increase in the TyG-BMI

Model 1 was without covariate adjustment.

Model 2 was adjusted for age.

Model 3 was adjusted for age, family income, smoking status, alcohol use, physical activity.

Model 4was adjusted for age, family income, smoking status, alcohol use, physical activity, diabetes, hypertension, high cholesterol, LDL, HDL, and HbA1c

TyG-BMI: triglyceride glucose-body mass index CVD: cardiovascular disease
